# Rolling Element Bearing Fault Diagnosis by Combining Adaptive Local Iterative Filtering, Modified Fuzzy Entropy and Support Vector Machine

**DOI:** 10.3390/e20120926

**Published:** 2018-12-04

**Authors:** Keheng Zhu, Liang Chen, Xiong Hu

**Affiliations:** 1 School of Logistics Engineering, Shanghai Maritime University, Shanghai 201306, China; 2 College of Energy and Power Engineering, Dalian University of Technology, Dalian 116023, China

**Keywords:** adaptive local iterative filtering, modified fuzzy entropy, SVM, rolling element bearing, fault diagnosis

## Abstract

A new fault feature extraction method for rolling element bearing is put forward in this paper based on the adaptive local iterative filtering (ALIF) algorithm and the modified fuzzy entropy. Due to the bearing vibration signals’ non-stationary and nonlinear characteristics, the ALIF method, which is a new approach for the analysis of the non-stationary signals, is used to decompose the original vibration signals into a series of mode components. Fuzzy entropy (FuzzyEn) is a nonlinear dynamic parameter for measuring the signals’ complexity. However, it only emphasizes the signals’ local characteristics while neglecting its global fluctuation. Considering the global fluctuation of bearing vibration signals will change with the bearing working condition varying, we modified the FuzzyEn. The modified FuzzyEn (MFuzzyEn) of the first few modes obtained by the ALIF is utilized to form the fault feature vectors. Subsequently, the corresponding feature vectors are input into the multi-class SVM classifier to accomplish the bearing fault identification automatically. The experimental analysis demonstrates that the presented ALIF-MFuzzyEn-SVM approach can effectively recognize the different fault categories and different levels of bearing fault severity.

## 1. Introduction

As a vital and widely used unit in the machinery system, rolling element bearings play a crucial role in the rotary machines. Bearing failure can cause enormous economic losses and can even be catastrophic [1,2]. Therefore, it is significant to develop an effective method for bearing fault diagnosis to guarantee the safe operation of the mechanical system. Over the last decades, most fault diagnosis approaches of rolling element bearing have been about vibration signal analysis, because rich dynamic information on machine health condition is contained in the signals generated by bearing vibration [3,4,5,6,7].

Usually, the bearing vibration signals will show characteristics of non-stationarity and non-linearity [8,9]. In recent years, various advanced time-frequency analysis techniques have been developed to process the non-stationary signals. As the most representative adaptive analysis method for non-stationary signals, the empirical mode decomposition (EMD) presented by Huang et al. [10] has attracted many researchers’ attention [8,11,12]. Although EMD can match the signal characteristic without a priori selection of any basis, it exhibits some shortcomings, such as a lack of mathematical foundation and mode mixing [13]. In order to overcome the limitations existing with EMD, some new adaptive mode decomposition methods have been developed, such as local mean decomposition (LMD) [14], empirical wavelet transform (EWT) [15] and variational mode decomposition (VMD) [16]. Liu et al. [17] used LMD to decompose bearing vibration signals effectively. However, LMD also suffers the shortcomings of mode mixing and the parameters of smoothing and step size need to be selected properly according to the signal characteristics. EWT and VMD are two variants of EMD, which have successful applications in the field of bearing fault diagnosis [18,19,20]. For the EWT method, the question of how to properly perform the spectrum segmentation is still a problem, while VMD has the requirement of predetermining the number of decomposition modes. Recently, the adaptive local iterative filtering (ALIF) method was developed by Cicone et al. [21] for analysis of non-stationary signals, which can produce completely data-driven decompositions and can suppress mode mixing [22,23]. Therefore, the ALIF approach is adopted in this study to process vibration signals of rolling element bearing.

After the vibration signals are decomposed by ALIF, the fault information needs to be extracted from the decomposition modes. Targeting the characteristics of the non-stationarity and non-linearity of vibration signals of rolling element bearing, many non-linear analysis parameters have been introduced to extract information on the bearing conditions. For the rolling element bearing, vibration signals of different states will exhibit diverse complexity. Hence, some entropy parameters have been investigated to diagnose the bearing faults. Therein, appropriate entropy (ApEn) and its improvement sample entropy (SampEn) as well as multi-scale entropy (MSE), hierarchical entropy (HE), were used to measure the complexity of bearing vibration signals. Through this, a good level of performance of fault feature extraction can be achieved [24,25,26]. Nevertheless, the similarity degree between vectors in SampEn is designed on the basis of Heaviside function whose boundary is rigid. However, the classes’ boundaries are ambiguous in the real applications, so it has difficulty in determining if an input pattern belongs to a class [27]. Therefore, the fuzzy entropy (FuzzyEn) algorithm was put forward in [27], in which exponential function replacing Heaviside function. Because of continuous boundaries of exponential functions, FuzzyEn is defined more accurately and shows a better statistical stability. On the basis of FuzzyEn, the multi-scale fuzzy entropy method was developed by Zheng et al. [28] and it was applied for bearing fault diagnosis. Li et al. [29] proposed a kind of improved multi-scale fuzzy entropy for the avoidance of inaccurate estimation of entropy values and used it to evaluate complexity of bearing vibration signals. However, FuzzyEn only emphasizes the signals’ local characteristics while neglecting its global characteristics because of the vector generalization of removing a local mean [30]. Considering that the global characteristics of vibration signals of rolling element bearing may vary as the bearing runs under different conditions, it may be unsuitable to assess the bearing vibration signals’ complexity by using the original FuzzyEn. Based on this consideration, in this paper, we modified the algorithm of FuzzyEn by removing the local mean used in the procedure of vector generalization, and the modified FuzzyEn was then adopted to acquire fault-related information from the decomposition modes obtained by the ALIF.

Naturally, after the fault features are extracted, an intelligent classifier is required to automatically identify the bearing fault categories as well as different levels of fault severities. Over the past decades, various pattern recognition methods have been applied in the field of mechanical fault diagnosis, among which support vector machines (SVM) [31,32] are the most commonly used ones. On the basis of statistical learning theory, SVM is suitable to address situations with small-quantity samples. At the same time, SVM has good generalization ability and can ensure the local and global optimal solution identical [33]. Because it has high recognition rate and superior generalization ability for a small quantity of samples, SVM is utilized in this paper to fulfill the fault identification of rolling element bearing.

To sum up, a novel fault diagnosis approach for rolling element bearing is proposed by combining ALIF, modified FuzzyEn and SVM in this study. The remainder of this paper is described as follows. The ALIF algorithm is given briefly in Section 2. Section 3 gives the introduction of the modified FuzzyEn. In Section 4, the fault diagnosis approach of rolling element bearing is put forward. Experimental validation of the presented approach is given in Section 5. Section 6 provides some conclusions finally.

## 2. Adaptive Local Iterative Filtering

The ALIF algorithm is the improvement of iterative filtering (IF) [34], which uses the same algorithm framework as the original EMD but computes the moving average of the signal through convolution using low pass filters [21]. There are two main differences between ALIF and IF. One is that ALIF calculates the filter length locally and adaptively. The other is that ALIF computes the moving mean of signals by means of the so-called Fokker–Planck filters, which are achieved as solutions of FP equations [21]. The ALIF algorithm is depicted as below. Given a signal f(x),xϵR, the moving average of the signal f(x) can be designated as:

(1) $$L_{\omega_{n},l_{n}}\left(f\right)\left(x\right)=\int_{-l_{n}\left(x\right)}^{l_{n}\left(x\right)}f\left(x+t\right)\omega_{n}\left(x,t\right)dt$$

where $\omega_{n}\left(x,t\right)$ is the filter at step *n* while $2l_{n}\left(x\right)$ is the mask length.

Assuming $f_{1}=f$, the fluctuation in $f_{n}$ can be captured by the operator which is defined as $S_{1,n}\left(f_{n}\right)=f_{n}-L_{\omega_{n},l_{n}}^{\left(1\right)}\left(f_{n}\right)=f_{n+1}$. Then the first mode can be obtained as $M_{1}=\underset{n\to \infty }{\lim}S_{1,n}\left(f_{n}\right)$. There are two loops in the ALIF algorithm: the inner loop and the outer loop. The inner loop extracts a single mode while the outer loop generates all the modes contained in a signal [21]. The iteration equation of the inner loop is denoted as:

(2) $$f_{n+1}\left(x\right)=f_{n}\left(x\right)-\int_{-l_{n}\left(x\right)}^{l_{n}\left(x\right)}f_{n}\left(x+t\right)\omega_{n}\left(x,t\right)dt$$

In real applications, *n* is limited based on a stopping criterion, which is defined as follows:

(3) $$SD=\frac{{\left\|M_{1,n}-M_{1,n-1}\right\|}_{2}}{{\left\|M_{1,n-1}\right\|}_{2}}$$

The inner iteration is stopped when the *SD* value reaches a certain threshold as recommended in literature [10,34]. The outer iteration stops when the remainder signal *r* becomes a trend signal, which indicates it has no more than one local extreme point. The remainder signal is calculated as $r=f-M_{1}-\cdots -M_{k-1}$. The description in detail about ALIF can be found in [21].

## 3. Modified Fuzzy Entropy

### 3.1. Fuzzy Entropy

SampEn adopts Heaviside function to assess the two vectors’ similarity degree while FuzzyEn uses fuzzy function for the similarity calculation. The computation procedures of FuzzyEn are presented as follows [27,35].

(1) For a sequence with length *N* {u(i):1≤i≤N}, construct the vectors of *m*-dimension $X_{i}^{m}$:
  (4) $$X_{i}^{m}=\left\{u\left(i\right),u\left(i+1\right),\cdots ,u\left(i+m-1\right)\right\}-u_{0}\left(i\right)1\leq i\leq N-m+1$$
  where $X_{i}^{m}$ stands for a new time series, being generalized by subtracting the mean of the *m* consecutive *u* values:
  (5) $$u_{0}\left(i\right)=m^{-1}\overset{m-1}{\underset{j=0}{\sum }}u\left(i+j\right)$$
(2) The distance between $X_{i}^{m}$ and $X_{j}^{m}$ is denoted as
  (6) $$d_{ij}^{m}=d\left[X_{i}^{m},X_{j}^{m}\right]=\underset{k\in \left[0,m-1\right]}{\max}|\left(u\left(i+k\right)-u_{0}\left(i\right)\right)-\left(u\left(j+k\right)-u_{0}\left(j\right)\right)|$$
(3) The similarity degree $D_{ij}^{m}$ can be computed by
  (7) $$D_{ij}^{m}=\mu \left(d_{ij}^{m},r\right)$$
(4) Denote $\varphi_{i}^{m}\left(r\right)$ as
  (8) $$\varphi_{i}^{m}\left(r\right)={\left(N-m-1\right)}^{-1}\overset{N-m}{\underset{j=1,j\neq i}{\sum }}D_{ij}^{m}$$
(5) The function $\varphi_{i}^{m}\left(r\right)$ is defined as
  (9) $$\varphi^{m}\left(r\right)={\left(N-m\right)}^{-1}\overset{N-m}{\underset{i=1}{\sum }}\varphi_{i}^{m}\left(r\right)$$
(6) Similarly, the $\varphi_{i}^{m+1}\left(r\right)$ is obtained by repeating the above steps
  (10) $$\varphi^{m+1}\left(r\right)={\left(N-m\right)}^{-1}\overset{N-m}{\underset{i=1}{\sum }}\varphi_{i}^{m+1}\left(r\right)$$
(7) Then define FuzzyEn of the sequence
  (11) $$FuzzyEn\left(m,r\right)=\underset{N\to \infty }{\lim}\left[ln\varphi^{m}\left(r\right)-ln\varphi^{m+1}\left(r\right)\right]$$
(8) Lastly, for a *N* with finite length, FuzzyEn could be calculated by
  (12) $$FuzzyEn\left(m,r,N\right)=ln\varphi^{m}\left(r\right)-ln\varphi^{m+1}\left(r\right)$$

The exponential function used in FuzzyEn was defined in [27] as

(13) $$\mu \left(d,r,n\right)=e^{-{\left(d/r\right)}^{n}}$$

### 3.2. Modified Fuzzy Entropy

One difference between FuzzyEn and SampEn is the computation of the vectors’ similarity degree. Another difference is the construction of *m*-dimensional vectors $X_{i}^{m}$, which are generalized by removing the mean of the segment of the time series defined by Equation (5). However, this implementation makes FuzzyEn focus only on the signals’ local characteristics while neglecting the corresponding global characteristics [30]. The global fluctuation in the bearing vibration signals may change with bearing states varying. Therefore, it may be not suitable to measure the bearing vibration signals’ complexity of using original FuzzyEn. Based on this consideration, the local mean is removed and the Equation (5) is modified as

(14) $$X_{i}^{m}=\left\{u\left(i\right),u\left(i+1\right),\cdots ,u\left(i+m-1\right)\right\}1\leq i\leq N-m+1$$

In this paper, the Equation (14) was utilized to calculate the fuzzy entropy values to evaluate the complexity of bearing vibration signals. By combining the modified FuzzyEn (MFuzzyEn) and ALIF, a new feature extraction approach of rolling element bearing is put forward by calculating the MFuzzyEn values over different modes obtained by ALIF.

### 3.3. Parameter Selection

Before the calculation of MFuzzyEn, four parameters need to be determined, viz. *m*, *r*, *N*, and *n*. The detailed reconstruction of the dynamic process is determined by the embedding dimension *m*. Normally, *m* is set to 2. The fuzzy entropy value depends less on the record length, so *N* is set to 4096 in this paper. The width of the fuzzy function boundary is decided by the parameter *r* while the boundary gradient is determined by the parameter *n*. According to previous study [25,27], *r* is chosen by 0.1–0.25 multiplying standard deviation (SD) and *n* should be small integers. Here, *r* = 0.2SD is selected while *n* = 2 is given.

## 4. The Proposed Bearing Fault Diagnosis Method

By integrating ALIF, modified FuzzyEn and SVM, a novel rolling element bearing fault diagnosis approach is presented as below:

(1) Vibration signals of rolling element bearing under different conditions are acquired by using an accelerometer.
(2) The ALIF algorithm is utilized to decompose the acquired bearing vibration signals and a series of mode components are obtained. The first several modes containing rich fault information are chosen for research.
(3) Calculate the MFuzzyEn of chosen components, and then the corresponding entropy value is treated as fault feature for reflecting working conditions of rolling element bearing.
(4) The obtained fault feature set is used for the training and testing of multi-class SVM classifier and fault recognition for rolling element bearing is completed automatically.

**Figure 1:**
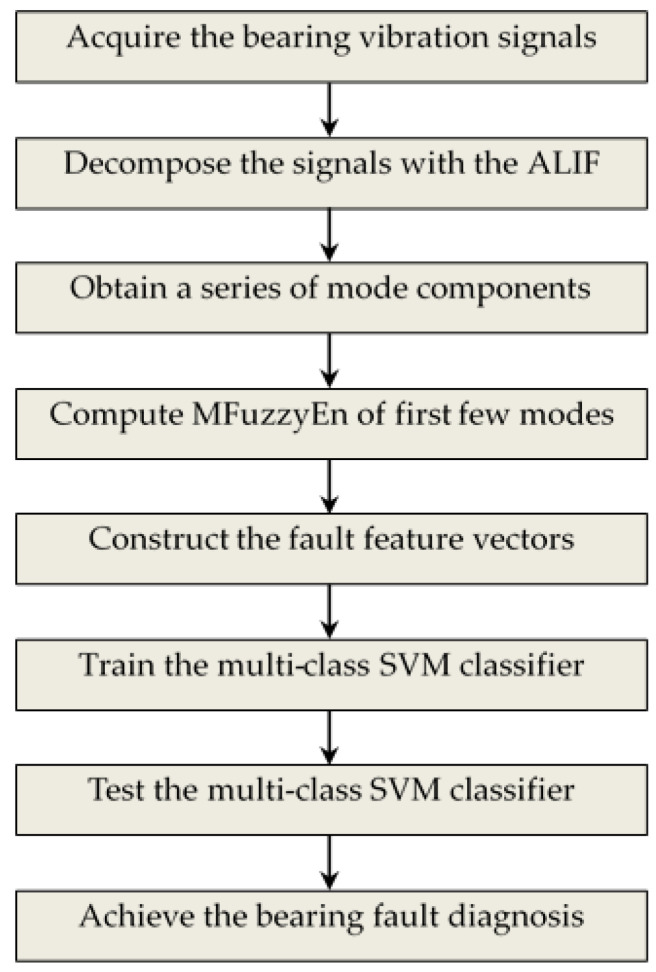
Flow chart of the proposed fault diagnosis method.

The flow chart of the proposed fault diagnosis method is illustrated in Figure 1.

## 5. Application

### 5.1. Experimental Data

**Figure 2:**
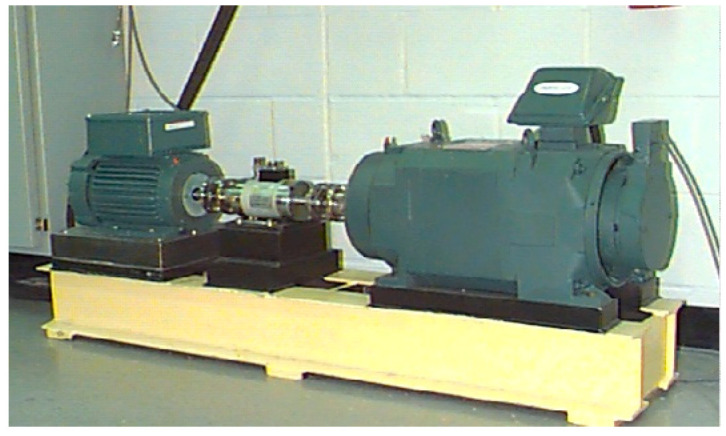
Picture of the experimental setup.

The vibration signals coming from the bearing data center of Case Western Reserve University [36] are used in this paper. The experimental data acquisition apparatus and description can be found in detail in the same literature. The experimental setup is shown in Figure 2. For each test bearing, three fault categories were simulated: inner race fault, outer race fault and rolling element fault. For each fault type, the fault diameters include 0.1778 mm, 0.3556 mm, 0.5334 mm as well as 0.7112 mm. An accelerometer mounted on the motor housing was adopted to acquire the vibration data at a sampling frequency of 12 kHz and the collected data is truncated into a 4096-point signal for further processing. The bearing vibration data was used for analysis when the load was 0 horsepower and the corresponding speed was 1797 rpm. The data description in detail is shown in Table 1.

### 5.2. Experimental Analysis

To validate the effectiveness of the proposed method, the analysis of the experimental data is carried out. The vibration signals contain three fault categories and different severities, thus the bearing fault diagnosis is actually a ten-class recognition problem. The data set is composed of 290 samples in total, and the length of each data sample is 4096. Among these 290 data samples, there are 14 samples of each category, In total 140 samples are chosen at random as training data while the remaining 150 are used as testing data.

**Figure 3:**
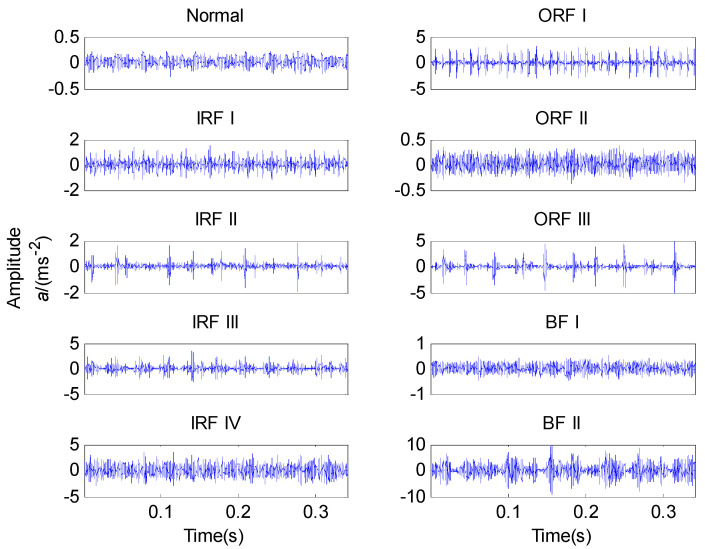
Vibration signals of ten bearing conditions.

The time waveforms of bearing vibration signals under ten working conditions are given in Figure 3. As is shown, it is hard to identify various bearing conditions based only on time domain waveforms. Therefore, it is essential to further process the original vibration signals. Due to the non-stationary and nonlinear characteristics of the vibration signals, the ALIF method is utilized to decompose them into a series of mode components. The state-related information is mainly hidden in the first several modes [22,23], so the first six modes are used to calculate the MFuzzyEn values, which form the feature vector.

**Figure 4:**
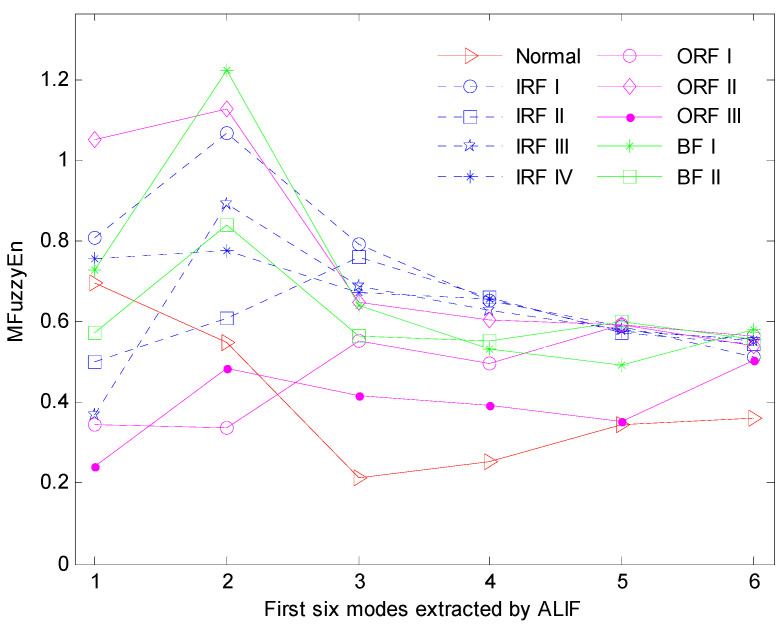
MFuzzyEn of the first six modes under ten bearing states.

**Figure 5:**
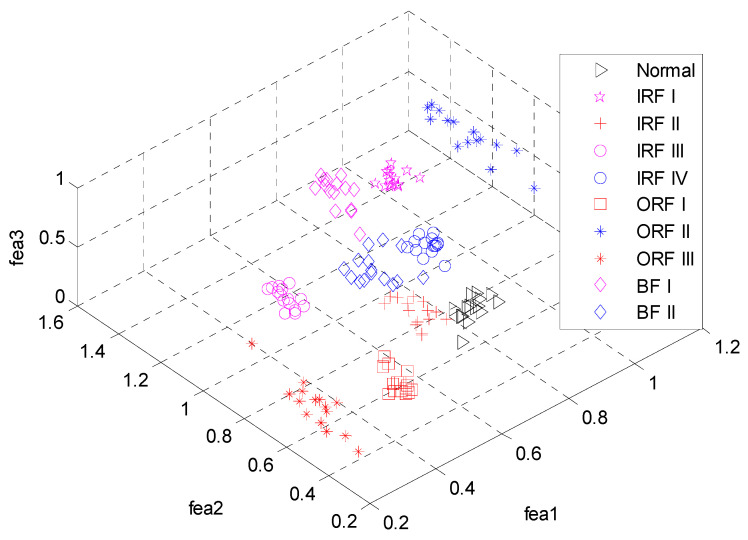
The first three features obtained by ALIF-MFuzzyEn.

**Figure 6:**
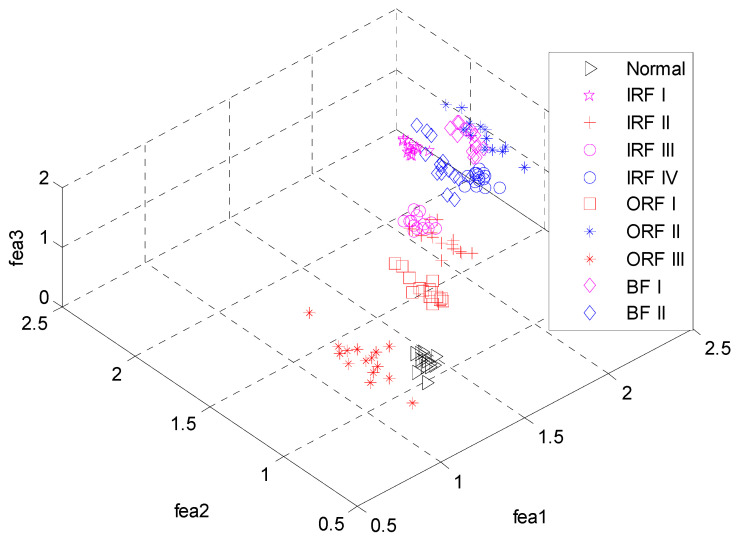
The first three features obtained by ALIF-FuzzyEn.

**Figure 7:**
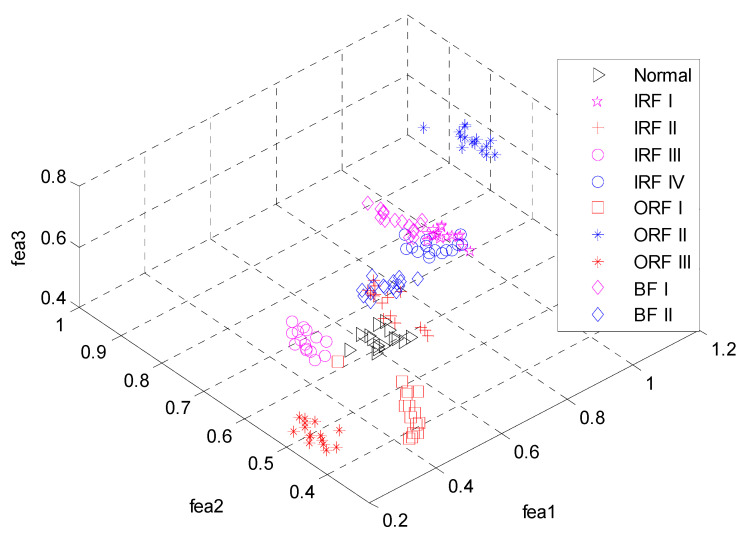
The first three features obtained by EMD-MFuzzyEn.

Consequently, 290 feature vectors can be obtained and the typical one for ten bearing conditions is illustrated in Figure 4. From Figure 2, we can see that vibration signals of different conditions have different entropy values over various modes. Especially for the first three modes, the entropy values present obvious distinction. To demonstrate the superiority of the feature extraction performance of the ALIF-MFuzzyEn over that of ALIF-FuzzyEn, the visualization of the first three features are correspondingly given in Figure 5 and Figure 6. It can be observed from Figure 5 that data points of the same working conditions are concentrated around one point and the data of different conditions are separated from each other. It can be seen from Figure 6 that there is overlapping between the data of some different conditions. This indicates that the ALIF-MFuzzyEn has better feature extraction ability than ALIF-FuzzyEn. For comparison purpose, the MFuzzyEn values of the first several modes obtained by using the EMD method (i.e., EMD-MFuzzyEn) are also computed and the first three features are shown in Figure 7. As shown in Figure 7, there also exists some data of different running states overlapping with each other. This comparison demonstrates that the feature extraction ability of ALIF-MFuzzyEn is superior to that of EMD-MFuzzyEn.

The above analysis shows the advantage of the presented ALIF-MFuzzyEn method over ALIF-FuzzyEn and EMD-MFuzzyEn. Therefore, the ALIF-MFuzzyEn is employed to form feature vectors and to train multi-class SVM, where the kernel of radial basis function (RBF) is chosen because of its merits [37]. For the RBF-SVM, there are two parameters needing to be chosen, which are the optimal kernel parameter and penalty parameter. In this paper, these two parameters will be obtained by using the five-fold cross-validation approach. After training, the feature vectors of test data can be identified by a trained SVM classifier.

**Figure 8:**
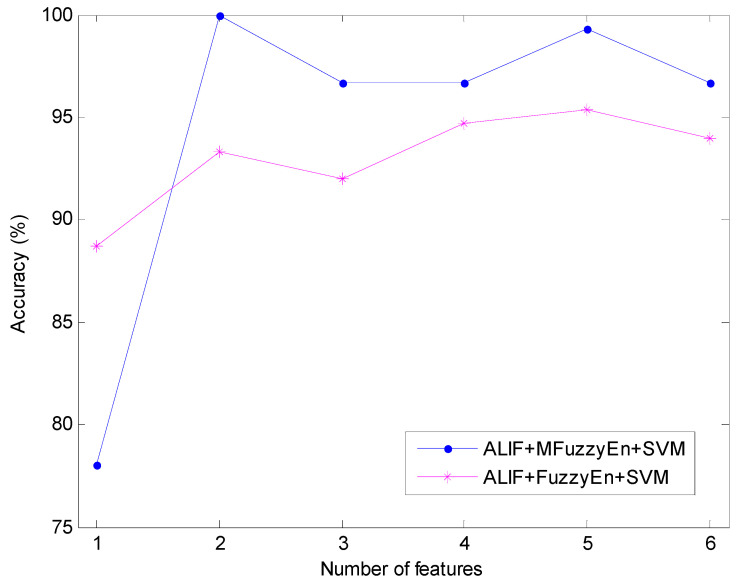
Classification accuracy comparison of MFuzzyEn and FuzzyEn with different number of features.

To contrast the recognition performance of ALIF-MFuzzyEn-SVM with that of ALIF-FuzzyEn-SVM, the feature vectors obtained by ALIF-FuzzyEn are also used for the training and testing of the SVM classifier. At the same time, in order for a comprehensive comparison, the effect of the number of used features is investigated. The corresponding classification results are described in Table 2. As it can be seen, the identification rate changes with the number of used features varying. The best classification rate of the ALIF-MFuzzyEn-SVM method can achieve 100% when the number of used features is two. On the contrary, the highest identification rate of the ALIF-FuzzyEn-SVM method is only 95.33% when the first five features are used for classification, with seven samples misclassified. To more clearly demonstrate the comparison between the aforementioned two methods, the classification accuracy comparison versus the number of used features is presented in Figure 8. We can observe from Figure 8 that all the recognition rates based on ALIF-MFuzzyEn-SVM are higher than those based on ALIF-FuzzyEn-SVM, except when the feature number equals one. Although the ALIF-FuzzyEn-SVM based accuracy is relatively high when the feature number is equal to one, it is just 88.67%, which is far below 100%. This comparison results verify that ALIF-MFuzzyEn-SVM has better classification performance than ALIF-MFuzzyEn-SVM.

**Figure 9:**
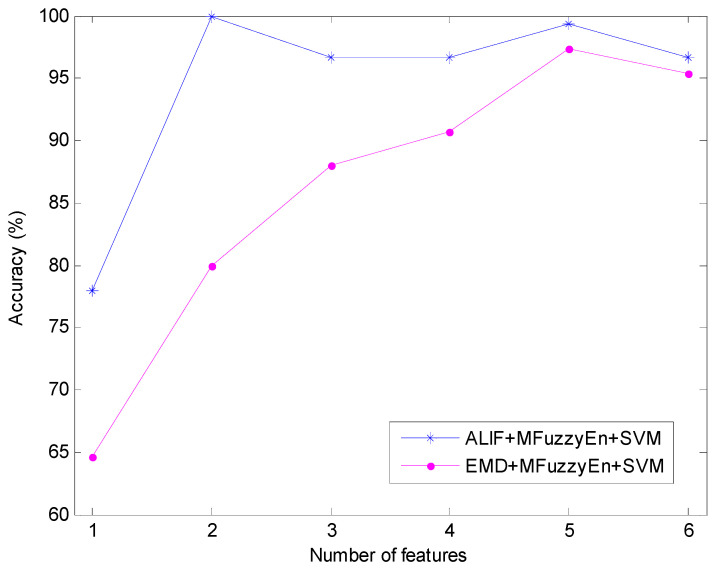
Classification accuracy comparison of ALIF and EMD with different number of features.

Moreover, the classification result of EMD-MFuzzyEn-SVM is also utilized for comparison with that of ALIF-MFuzzyEn-SVM. The identification results of these two methods versus different feature numbers are shown in Figure 9, from which it can be seen that all the identification rates of EMD-MFuzzyEn-SVM are lower than those of ALIF-MFuzzyEn-SVM. The best classification accuracy based on EMD-MFuzzyEn-SVM is 97.33%, lower than that of ALIF-MFuzzyEn-SVM, that is 100%. The above results show the advantage of ALIF-MFuzzyEn-SVM over EMD-MFuzzyEn-SVM in classification performance and further verify the effectiveness of the developed ALIF-MFuzzyEn-SVM approach.

## 6. Conclusions

A new rolling element bearing fault diagnosis approach is put forward in this paper by combining adaptive local iterative filtering (ALIF), modified fuzzy entropy (MFuzzyEn) and support vector machine (SVM). The ALIF algorithm is utilized to decompose bearing vibration signals and then a series of modes are obtained. The MFuzzyEn values of the first few modes are computed to form fault feature vectors, which are then input into the SVM classifier to realize fault pattern recognition. For the purpose of comparison, FuzzyEn is also used to analyze the experimental signals, and the comparison results indicate that ALIF-MFuzzyEn has better feature extraction ability than ALIF-FuzzyEn. Furthermore, the effectiveness of ALIF is compared with that of EMD, which shows that the presented ALIF-MFuzzyEn-SVM approach can obtain higher accuracy than EMD-MFuzzyEn-SVM. The experimental analysis demonstrates that the developed method based on ALIF, MFuzzyEn and SVM can effectively identify bearing fault categories and various levels of severities. However, the proposed approach only considers the vibration data under the same bearing speed. The effect of the speed changes on the fault identification should be investigated in the future.

## Figures and Tables

**Table 1:** Description of experimental data.

Bearing State	Fault Diameter (mm)	Label of Classification	Bearing State	Fault Diameter (mm)	Label of Classification
Normal	0	1	ORF I	0.1778	6
IRF I	0.1778	2	ORF II	0.3556	7
IRF II	0.3556	3	ORF III	0.5334	8
IRF III	0.5334	4	BF I	0.1778	9
IRF IV	0.7112	5	BF II	0.7112	10

**Table 2:** Classification results of testing data based on the features extracted by FuzzyEn and MFuzzyEn with different number of features.

Used Features	ALIF + MFuzzyEn + SVM		ALIF + FuzzyEn + SVM	
	The Number of Misclassified Data	Accuracy (%)	The Number of Misclassified Data	Accuracy (%)
First 1	33	78	17	88.67
First 2	0	100	10	93.33
First 3	5	96.67	12	92
First 4	5	96.67	8	94.67
First 5	1	99.33	7	95.33
First 6	5	96.67	9	94
